# Identification and Characterization of a Novel Hyperthermostable Bifunctional Cellobiohydrolase- Xylanase Enzyme for Synergistic Effect With Commercial Cellulase on Pretreated Wheat Straw Degradation

**DOI:** 10.3389/fbioe.2020.00296

**Published:** 2020-04-09

**Authors:** Chao Han, Ruirui Yang, Yanxu Sun, Mengyu Liu, Lifan Zhou, Duochuan Li

**Affiliations:** Key Laboratory of Agricultural Microbiology, College of Plant Protection, Shandong Agricultural University, Tai’an, China

**Keywords:** bifunctional, thermostable, cellobiohydrolase, xylanase, synergism, pretreated corn stover

## Abstract

The novel cellobiohydrolase gene *ctcel7* was identified from *Chaetomium thermophilum*, and its recombinant protein CtCel7, a member of glycoside hydrolase family 7, was heterologously expressed in *Pichia pastoris* and biochemically characterized. Compared with commercial hydrolases, purified CtCel7 exhibited superior bifunctional cellobiohydrolase and xylanase activities against microcrystalline cellulose and xylan, respectively, under optimal conditions of 60°C and pH 4.0. Moreover, CtCel7 displayed remarkable thermostability with over 90% residual activity after heat (60°C) treatment for 180 min. CtCel7 was insensitive to most detected cations and reagents and preferentially cleaved the β-1,4-glycosidic bond to generate oligosaccharides through the continuous saccharification of lignocellulosic substrates, which are crucial for various practical applications. Notably, the hydrolysis effect of a commercial cellulase cocktail on pretreated wheat straw was substantively improved by its combination with CtCel7. Taken together, these excellent properties distinguish CtCel7 as a robust candidate for the biotechnological production of biofuels and biobased chemicals.

## Introduction

Lignocellulosic biomass is the most abundant renewable resource in nature and represents a promising feedstock for the agricultural, biochemical and biofuel industries (Liu et al., 2019). Cellulose and hemicellulose are the primary components of lignocellulosic biomass with mass ratios of 35–50% and 20–30%, respectively (Penttilä et al., 2018). Due to the structural complexity of lignocellulosic biomass, its efficient enzymatic depolymerization necessitates the synergistic actions of a diverse set of glycoside hydrolases (GHs), particularly cellulase and xylanase. Because multifunctional glycoside hydrolases with excellent activity are favorable for carbohydrate conversion (Cao et al., 2018; Wang et al., 2019), much effort has been devoted to the bioprospecting of these potent catalysts to allow their application in industrial fields. Although many multifunctional GHs have been identified (Yang et al., 2017; Tan et al., 2018; Liu et al., 2019), an inherent enzymatic feature of significant thermostability, which is preferred for practical production, has rarely been reported, especially for a thermostable bifunctional cellobiohydrolase capable of xylan hydrolysis.

In general, the optimal activity of thermostable glycoside hydrolases is observed at temperatures exceeding 50°C, which is compatible with actual preheat treatment procedures and makes the enzyme-catalytic process cost-competitive (Boyce and Walsh, 2015). Therefore, a realistic biorefinery of lignocellulosic saccharification is, as a rule, implemented under elevated temperatures and has distinct advantages, including promoting biomass disorganization, increasing substrate solubility, lowering viscosity, and preventing microbial contamination (Bhalla et al., 2013; Kadowaki et al., 2018). In this sense, potent thermostable glycoside hydrolases endowed with pronounced activity at high temperatures serve as valuable biocatalysts that can accelerate the catalytic process, shorten the reaction period and reduce the enzyme dosage (Liu et al., 2019). As a consequence, the identification and utilization of thermostable glycoside hydrolases undoubtedly remain important research topics. Currently, thermophiles are often regarded as exploitable sources of industrially relevant thermostable enzymes (Turner et al., 2007). *Chaetomium thermophilum* is a representative thermophilic fungus and secretes biomass-degrading enzymes belonging to diverse glycoside hydrolase (GH) families according to the classification guidelines indicated in the Carbohydrate Active enZYmes (CAZY) database (Lombard et al., 2014). Recent advances have identified thermostable glycoside hydrolases from *C. thermophilum* with extraordinary activity and thermostability at relatively high temperatures. These hydrolases include a GH6 cellobiohydrolase (CtCel6) (Zhou Q.Z. et al., 2017), a GH7 endoglucanase (CTendo7) (Hua et al., 2018), a GH45 endoglucanase (CTendo45) (Zhou Q. et al., 2017) and four GH11 xylanases (Ct Xyn11A, CTX, xyn698, and xyn669) (Hakulinen et al., 2003; Mäntylä et al., 2007; Ghaffar et al., 2011).

In the present study, a novel GH7 family glycoside hydrolase (CtCel7) from *C. thermophilum* that possesses prominent bifunctional cellobiohydrolase-xylanase activity and considerable thermostability was cloned, functionally expressed and biochemically characterized. Furthermore, the hydrolysis products from multiple lignocellulosic materials and the synergistic action of CtCel7 combined with a commercial cellulase cocktail on pretreated wheat straw were also evaluated.

## Materials and Methods

### Microbial Strains, Vectors and Chemical Reagents

The *C. thermophilum* strain CT2 was isolated from bovine feces at Tengchong (Yun’nan, China) and deposited in the publicly accessible China General Microbiological Culture Collection (CGMCC) under the registration number 3.17990. *Escherichia coli* Trans1-T1 (TransGen, Beijing, China) was used for gene sequencing and plasmid propagation. *Pichia pastoris* GS115 and the *Pichia*-secreted expression vector pPIC9K (Invitrogen, Carlsbad, CA, United States) were used for recombinant protein production. The purity of all the chemicals was of reagent grade.

### *In Silico* Sequence and Structural Analysis

Genome information of *C. thermophilum*^1^ was employed to search for the target gene *ctcel7*. The protein molecular weight and pI value were calculated using the online ProtParam program^2^. The signal peptide was predicted using the SignalP 4.1 server^3^. The NetNGlyc 1.0 Server^4^ and NetOGlyc 4.0 Server^5^ were used for glycosylation site analysis (Hua et al., 2018). Sequence similarity analysis was performed through a BLAST search^6^. Multiple sequence alignment was performed using ClustalX2 and ESPript 3.0 (Wu et al., 2018). The homology-modeling structure was constructed using PyMOL software based on an identified *Myceliophthora thermophila* GH7 thermostable cellobiohydrolase (MtCel7A, PDB: 5W11) (Kadowaki et al., 2018), which shares 82.3% amino-acid identity with CtCel7.

### Gene Cloning and Transformation

After induced culturing with microcrystalline cellulose (Avicel) as the sole carbon source at 50°C for 3 days, total RNA was extracted from the mycelia of *C. thermophilum* using the TRIzol reagent (Invitrogen). RT-PCR was performed to amplify the putative *ctcel7* cellobiohydrolase gene (GenBank accession no. AY861347) using the following primers: forward primer, 5′-CCGGAATTCCAGCAGGCTTGCTCCCT-3′; reverse primer, 5′-TTGCGGCCGCTTA*GTGGTGGTGGTGGTGGTG*CAGGCAC TGGCTGT-3′ (italics denote the C-terminal hexahistidine tag, and underlined letters indicate the *Eco*RI and *Not*I restriction sites).

The purified PCR product and pPIC9K vector were treated with *Eco*RI and *Not*I (Fermentas, MD, United States) and ligated using T4 DNA ligase (Fermentas). The recombinant plasmid pPIC9K/*ctcel7* was linearized with *Sac*I and transformed into *P. pastoris* GS115. The transformants were seeded onto minimal dextrose (MD) and minimal methanol (MM) plates and were then screened on yeast peptone dextrose (YPD) agar medium supplemented with different concentrations of G418 (geneticin; Sangon, Shanghai, China) for the selection of multicopy integrants (Zhou Q. et al., 2017).

### Expression and Purification of Recombinant CtCel7

Methanol-induced enzyme expression was performed with shaking cultivation (200 rpm, 28°C) for 7 days according to the *Pichia* Expression Kit (Invitrogen). Subsequently, the cell-free supernatant was harvested by centrifugation (8,000 rpm, 20 min, 4°C) and adjusted with ammonium sulfate (80% saturation) at 4°C overnight (Han C. et al., 2018). The obtained precipitate was then dissolved in phosphate buffer solution (PBS, 20 mM, pH 7.4). After dialysis with PBS, the crude extract was centrifuged (8,000 rpm, 20 min, 4°C), and the supernatant was collected. Afterward, the crude enzyme was purified using a HisTrap HP column (GE Healthcare, Buckinghamshire, United Kingdom) equilibrated with binding buffer (300 mM NaCl, 20 mM sodium phosphate at pH 7.4, and 30 mM imidazole) and eluted with elution buffer (300 mM NaCl, 20 mM sodium phosphate at pH 7.4, and 250 mM imidazole) (Peng et al., 2016). Fractions were collected and dialyzed with 20 mM PBS (pH 7.4) to remove excess imidazole. The purified enzyme, termed CtCel7, was preserved and used for subsequent assays.

The protein concentration was determined using a Pierce BCA Protein Assay Kit (Thermo Scientific, MA, United States). Sodium dodecyl sulfate-polyacrylamide gel electrophoresis (SDS-PAGE) was performed to determine the purity and molecular mass of the protein through Coomassie Blue R-250 (Sigma-Aldrich, MO, United States) staining and using a Pierce Glycoprotein Staining Kit (Thermo Scientific), respectively (Hua et al., 2018). For zymogram analysis, the purified CtCel7 was run on a 12% (w/v) SDS-PAGE gel under nondenaturing conditions. The gel was then overlaid with 0.5% (w/v) β-glucan and 1% (w/v) xylan dissolved in sodium acetate buffer (200 mM, pH 5.0), respectively, as described previously (Phadtare et al., 2017).

### Enzyme Assay

The 3,5-dinitrosalicylic acid (DNS) method was employed for the detection of the cellulase and xylanase activities of the enzyme using barley β-D-glucan (Sigma-Aldrich) and beech wood xylan (Sigma-Aldrich) as the substrates, respectively, after 30 min of incubation at 60°C and pH 4.0. Standard curves were quantified with D-glucose and D-xylose, respectively, as described by Miller (1959). One international unit (IU) of enzymatic activity is defined as the amount of enzyme capable of releasing 1 μmol of reducing sugars per minute (Hua et al., 2018). Each experiment was performed in triplicate.

### Effect of pH and Temperature on Enzyme Activity

The optimal pH value for enzymatic activity was evaluated at 60°C in various 200 mM buffer solutions, including sodium acetate buffer (pH 3.0-6.0), sodium phosphate buffer (pH 6.0-8.0) and Tris–HCl buffer (pH 8.0-11.0). The optimal temperature within the range of 30–90°C was determined at the optimal pH (Lee et al., 2018).

### Thermostability Assay

The thermostability of the enzyme was determined by preincubating the enzyme at 60–90°C for multiple time periods ranging from 30 to 180 min. The residual activities were then assessed using 0.2% (w/v) barley β-D-glucan (Sigma-Aldrich) and 0.5% (w/v) beech wood xylan (Sigma-Aldrich) as the substrates. Non-preincubated enzyme was used as a control.

### Effect of Cations and Chemicals on Enzyme Activity

The effect of 11 kinds of metal salts (MnCl_2_, CaCl_2_, MgCl_2_, CoCl_2_, SrCl_2_, NiSO_4_, ZnCl_2_, CrCl_3_, AlCl_3_, CuSO_4_, and FeCl_3_) on the cellulase and xylanase activities of CtCel7 were detected at final ion concentrations of 1 and 5 mM, respectively (Yang et al., 2017; Wang et al., 2019). The influence of eight kinds of chemical reagents at different concentrations on the enzymatic activities was also monitored. Tween-20, Triton X-100, polyethylene glycol (PEG-600), and sodium dodecyl sulfate (SDS) were used as surfactants. Methanol, ethanol, and isopropanol are used as organic solvents. Ethylenediaminetetraacetic acid (EDTA) is used as a metal chelator. These metal salts and chemical reagents are commonly present in industrial practices and may have impacts on enzymatic activities (Boyce and Walsh, 2015; Wang et al., 2019). Thus, the cation and chemical resistance of CtCel7 is an evaluation index for its industrial applications, such as environmental waste treatment, functional oligosaccharide extraction and bioethanol production (Bajaj and Mahajan, 2019; Patel et al., 2019; Yang et al., 2019). The enzyme in the absence of metal ions or reagents was used as a control (Jeon et al., 2016).

### Substrate Specificity

A panel of polysaccharide substrates with different glycosidic bonds was applied to investigate the substrate preferences of the purified CtCel7 after incubation for 30 min at pH 4.0 and 60°C. The investigated substrates included sodium carboxymethyl cellulose (CMC-Na; 400–800 centipoise), xylan from beechwood, β-D-glucan from barley, amylose from potato, D-galacto-D-mannan from *Ceratonia silique*, chitin from shrimp shells, amylopectin from maize (all Sigma-Aldrich), (+)-arabinogalactan from larch wood (Tokyo Chemical Industry, Tokyo, Japan), sucrose (Solarbio Science and Technology, Beijing, China), filter paper (No. 1; Whatman, Chiltern, United Kingdom), and lichenin from Icelandic moss (Megazyme, Chicago, IL, United States). Phosphoric acid swollen cellulose (PASC) was prepared from Avicel (Sigma-Aldrich) according to a method previously described by Wood (1988). PASC, CMC-Na, amylose, mannan and sucrose were used at a concentration of 1% (w/v). The concentration of xylan and amylopectin was 0.5% (w/v), whereas the concentration of glucan, lichenin and arabinogalactan was 0.2% (w/v). Each reaction solution included 12 mg of chitin and filter paper. Commercial GH7 cellobiohydrolase I (Megazyme) and GH11 β-1,4-xylanase (Sigma-Aldrich), both from *Trichoderma longibrachiatum* and abbreviated TlCBHI and TlXyn, respectively, were employed as controls and incubated for 30 min under optimal conditions (pH 4.5 at 40 and 30°C, respectively).

### Kinetic Parameters

Equivalent amounts of the CtCel7 protein were used to hydrolyze varying concentrations of glucan (0.1–1 mg/ml) and xylan (0.2–2 mg/ml). The reactions were performed under standard conditions as mentioned above. The kinetic parameters were calculated according to the Michaelis-Menten equation as previously described (Carlin et al., 2016).

### Hydrolysis Product Analysis

The CtCel7 hydrolysis product profile was qualitatively determined by thin layer chromatography (TLC) after incubation for various time periods (10, 30, 60, 120, and 180 min) with glucan, xylan, PASC, and filter paper as the substrates (Cheng et al., 2012). The hydrolysis products at various periods and the oligosaccharide mixture standard (Gentaur, Kampenhout, Belgium) were individually spotted on a silica plate (type 60 F254; Merck, Germany), and the plate was subsequently developed with an ethyl acetate/methanol/water/acetic acid (4:2:1:0.5, v/v/v/v) solvent system in a covered chamber (Hua et al., 2018). The results were visualized by dipping into a solution containing 2% (w/v) N-phenylaniline, 2% (v/v) phenylamine, and 85% (v/v) phosphoric acid in acetone, followed by heating at 85°C for 15 min (Li W. et al., 2018). Commercial TlCBHI and TlXyn were used as controls under their individual optimal reaction conditions for 180 min. The relative proportions of different oligosaccharide products were evaluated through gray-value analysis of the TLC results using ImageJ software.

### Hydrolysis of Pretreated Wheat Straw

Wheat straw was gathered from Tai’an (Shandong, China) and pretreated with NaClO_2_/3% dilute sulfuric acid (1:0.5, v/v) at 70°C for 4 h as previously described (Qi et al., 2010). A typical commercial cellulase cocktail derived from *Trichoderma reesei* (cellulase for simplicity, Sigma-Aldrich) was used to investigate the synergistic action of CtCel7. The hydrolysis effects of CtCel7 and cellulase on pretreated wheat straw (PWS) were analyzed under optimal reaction conditions (pH 4.0 at 60°C for CtCel7; and pH 5.0 at 37°C for cellulase) in an orbital shaker at a speed of 250 rpm for different time intervals. Synergistic hydrolysis was conducted under the optimal reaction conditions of CtCel7 and cellulase. In a total reaction solution of 400 μl, the initial concentration of PCS was 30 mg/ml, and the enzyme loading concentration of CtCel7 and cellulase was 0.2 mg/ml (Wang et al., 2019). The supernatant was collected after filtering through a 0.45 μm membrane, and the amount of released reducing sugars was assessed using the DNS method as mentioned above.

## Results and Discussion

### Sequence Analysis of CtCel7

**FIGURE 1:**
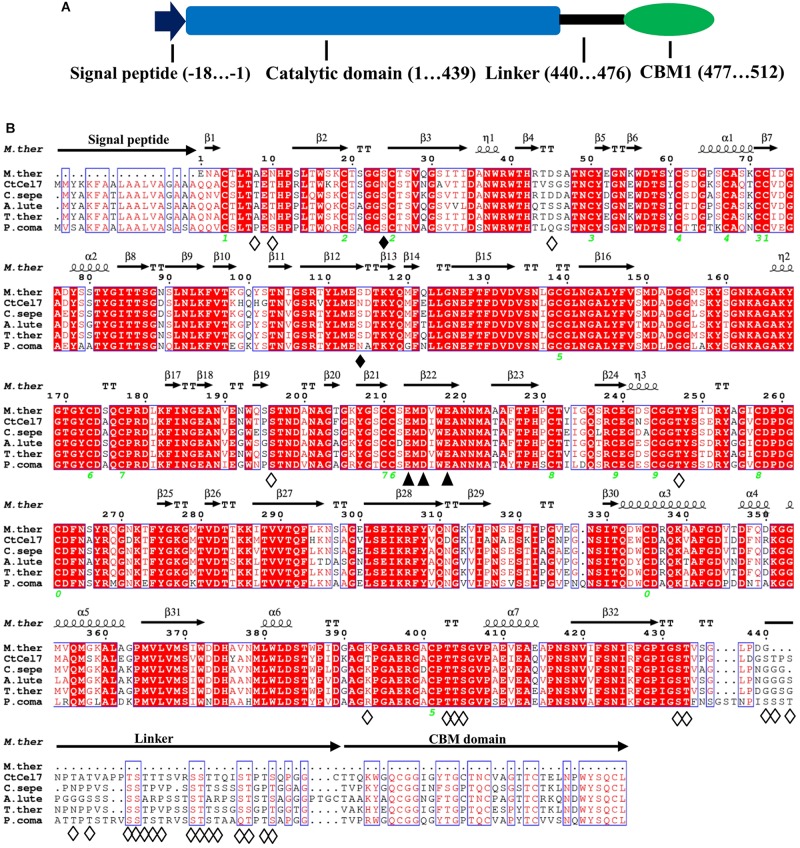
**(A)** Schematic representation of the primary sequence of the multimodular CtCel7 protein. **(B)** Multiple sequence alignment of CtCel7 with selected GH7 cellobiohydrolases, including *Myceliophthora thermophila* (PDB: 5W11, 82.3% identity), *Podospora comata* (VBB76653, 72.31% identity), *Achaetomium luteum* (AGV05131, 74.15% identity), *Thermothelomyces thermophilus* (XP_003660789, 75.80% identity), and *Corynascus sepedonium* (AGV05125, 76.56% identity). The strictly conserved residues are highlighted by a red background. Regions consisting of conserved, highly similar residues are framed by thin-lined boxes. The secondary structural elements of CtCel7 are represented above the aligned sequences. The green number indicates the location of disulfide bonds. The catalytic triad residues Glu213, Asp215 and Glu218 are denoted with closed triangles. The solid and hollow diamonds specify the predicted N-glycosylation sites and O-glycosylation sites, respectively. The alignment was performed using ClustalX2 and ESPript 3.0.

The putative *C. thermophilum* cellobiohydrolase-encoding gene (*ctcel7*) contains a 1593-bp open reading frame (ORF) encoding a 530-amino-acid product that exhibits 100% identity with an unstudied exoglucanase-like protein (GenBank accession no. XP_006693547) from *C. thermophilum* var. *thermophilum* DSM 1495. The encoded CtCel7 protein has a calculated molecular mass of 56 kDa and a pI value of 5.04. The N-terminal signal peptide suggests that the multimodular CtCel7 is secreted extracellularly, and the signal peptide is followed by a typical GH7 catalytic domain (CD), a Ser/Thr-rich linker region and a C-terminal CBM1 (Figure 1A). Two potential N-glycosylation sites and 11 O-glycosylation sites were predicted in the CtCel7_CD region, which implies that CtCel7 is glycosylated (Figure 1B).

**FIGURE 2:**
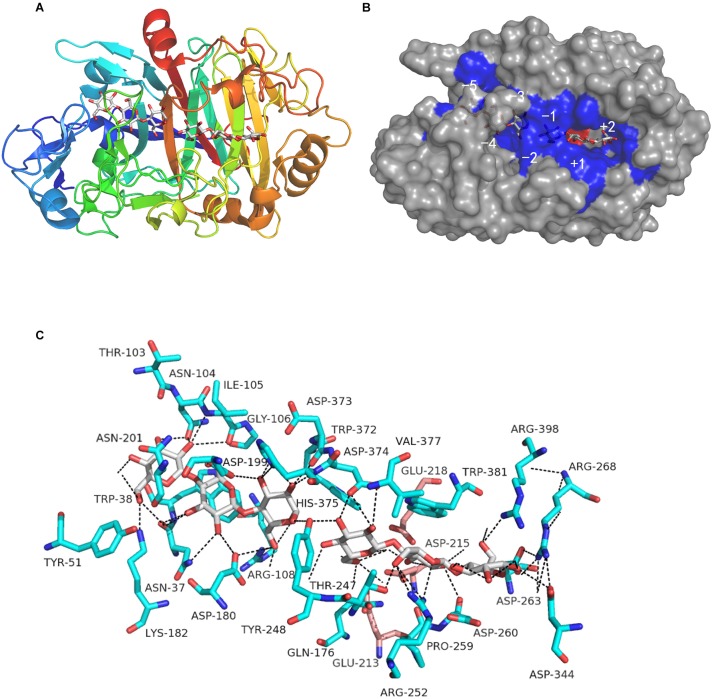
Predicted three-dimensional structure of the catalytic domain of the CtCel7 protein obtained using the *Myceliophthora thermophila* thermostable cellobiohydrolase Cel7A as the template (PDB: 5W11). **(A)** Tertiary structure of CtCel7 in complex with cellotriose molecules shown in stick format. The color gradient shows the sequence from the N-terminus (blue) to the C-terminus (red). **(B)** The solvent-accessible surface of CtCel7 occupying the subsites is indicated. The amino acids that directly interact with ligands are shaded blue, and the catalytic acids are marked in red. **(C)** The residue positions that make direct polar contact with the two cellotriose molecules are marked in blue. In particular, the putative catalytic triad residues Glu213, Asp215, and Glu218 are presented as pink sticks. Hydrogen bonds are presented as black dashed lines. All of the structural diagrams were drawn using PyMOL software.

The BLAST search indicated a high degree of identity with other reported GH7 cellobiohydrolases. From a structural perspective, the entire CtCel7_CD region houses the classical curved β-sandwich constructed with two largely antiparallel β-sheets in an equatorial elongated substrate-binding tunnel, which is in accordance with the archetypal feature of GH7 CBH structures (Figure 2A; Momeni et al., 2014; Borisova et al., 2015). This predominant structural feature allows CtCel7 to act along cellulose chains without affecting the substrate configuration and clip off numerous cellobiose units before detaching from the substrate (Kadowaki et al., 2018), which is indispensable to its prominent hydrolysis efficiency on highly crystalline cellulose (Payne et al., 2015; Taylor et al., 2018). Combined with the homologous sequence alignment, the results identified the catalytic triad residues Glu213 (nucleophile), Asp215 and Glu218 (acid/base) in CtCel7 and revealed that these are embedded in the binding tunnel, where two cellotriose molecules (−5 to −3 and −1 to +2 subsites, respectively) are involved in coordinated interactions with adjacent residues located within 5 Å via hydrogen bonding (Figures 2B,C). Nonetheless, no ligand is evident at the −1 subsite.

### Cloning, Expression and Purification of the Recombinant CtCel7 Protein

**FIGURE 3:**
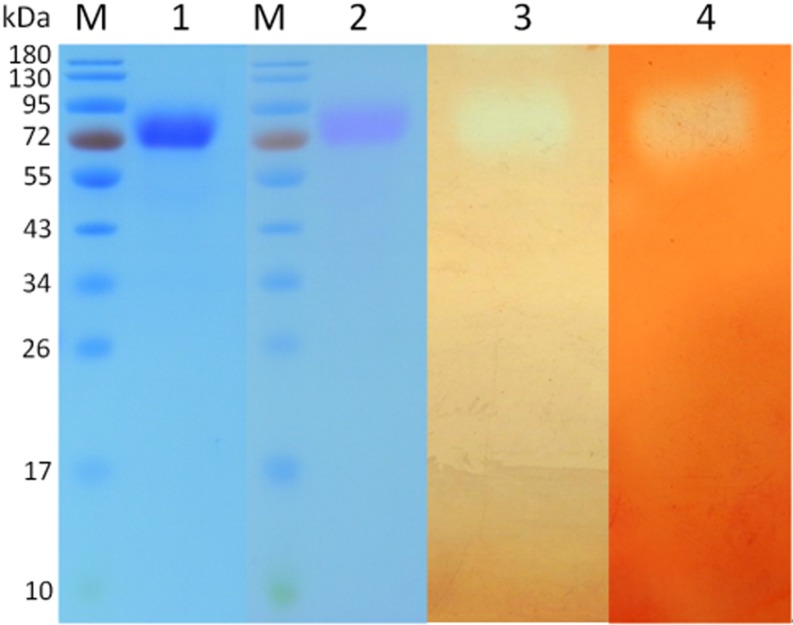
SDS-PAGE analysis and zymogram of the purified recombinant CtCel7 enzyme. Lane M, molecular mass markers; lane 1, Coomassie brilliant blue staining; lane 2, glycoprotein staining; lane 3, zone of β-D-glucan hydrolysis in the zymogram; and lane 4, zone of beechwood xylan hydrolysis in the zymogram.

To evaluate the enzymatic properties of CtCel7, the putative *C. thermophilum* CBH-encoding *ctcel7* gene was amplified by RT-PCR and heterologously expressed in *P. pastoris*. The recombinant CtCel7 was purified to a purification fold of 5.1 and a recovery yield of 62.4% after HisTrap HP (Table 1). The purified protein CtCel7 was confirmed by MALDI-TOF/TOF (Supplementary Figure S1) and SDS-PAGE (Figure 3). As estimated from the SDS-PAGE results, the apparent homogeneity had a higher molecular weight (approximately 73 kDa) than the predicted homogeneity (56 kDa), which can most likely be ascribed to glycosylation (Hua et al., 2018; Kadowaki et al., 2018). In addition, the glycoprotein staining analysis corroborated that CtCel7 was a glycoprotein (Figure 3). In the zymogram, purified CtCel7 displayed transparent zones at the concordant molecular mass, indicating the distinct enzymatic decompositions of glucan and xylan (Figure 3).

**TABLE 1:** Purification of recombinant CtCel7 heterologously expressed in *Pichia pastoris*.

	Cellulase activity (IU/mg)	Xylanase activity (IU/mg)	Total protein (mg)	Purification Fold	Yield (%)
Cell-free supernatant	0.38 ± 0.05	0.29 ± 0.04	502.3 ± 35.1	1.0	100
Ammonium sulfate	1.06 ± 0.12	0.81 ± 0.06	419.9 ± 28.2	2.8	83.6
HisTrap HP	1.94 ± 0.16	1.49 ± 0.11	313.4 ± 17.3	5.1	62.4

*Activities of cellulase and xylanase were measured at 60°C and pH 4.0 after 30 min of incubation using β-D-glucan and xylan as substrates, respectively. Values are the means ± SD of three replicates.*

### Optimal Reaction Conditions for Enzymatic Hydrolysis

**FIGURE 4:**
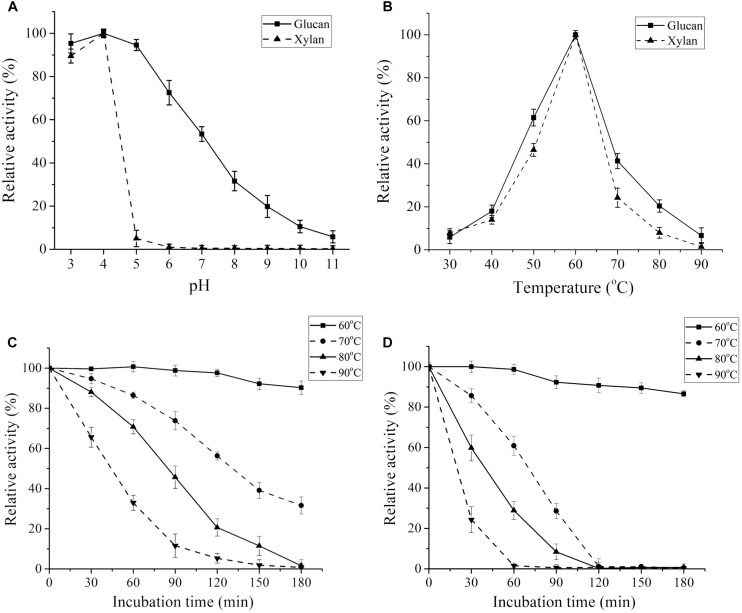
Enzymatic profiles of the purified recombinant CtCel7 bifunctional enzyme. The optimal pH **(A)** and temperature **(B)** of CtCel7 were assessed using glucan and xylan as the substrates. The thermostability of the enzyme was determined using glucan **(C)** and xylan **(D)** as the substrates. The activity of the unheated sample was set to 100%, and the relative hydrolytic activity at each time point is presented as a percentage. The values are the means ± SDs from three replicates.

The effects of temperature and pH on enzymatic activity were assessed using glucan and xylan as the substrates, respectively. The maximum cellulase activity of CtCel7 appeared at pH 4.0, which indicated that the enzyme exhibited an obvious preference for slightly acidic conditions (Figure 4A), and the optimal temperature was 60°C (Figure 4B). Consistently, the optimal conditions for CtCel7-xylanase activity were pH 4.0 and 60°C (Figures 4A,B). The concordant optimal reaction conditions for the bifunctional cellobiohydrolase and xylanase activities of CtCel7 are presumably related to the single substrate-binding tunnel threaded through the CtCel7_CD region (Figures 2A,B; Tan et al., 2018).

### Thermal Properties of CtCel7

Thermostability assays determined that CtCel7 adapted well to a temperature of 60°C, as indicated by the finding that the enzyme retained its full activity against glucan after 120 min of incubation at this temperature (Figure 4C). Additionally, more than approximately 90% residual activity was detected when the incubation time was prolonged to 180 min. During incubation at 70°C or higher temperatures for a relatively long preheating period, the cellulase activity was gradually reduced. A similar heat stability curve for CtCel7 xylanase activity was obtained, as illustrated in Figure 4D. These results indicate that CtCel7 has considerable thermal stability.

From a practical standpoint, thermostable GHs have intrinsic superiority over their mesophilic counterparts because high temperatures are necessary for heat-treatment processes in many biotechnological industries (Bhalla et al., 2013). Consequently, thermostable GHs with cellulase and xylanase activities would be more favorable for the bioconversion of lignocellulosic biomass by improving the hydrolytic performance, strengthening the cost-effectiveness of the process and expanding the scope of application (Wang et al., 2019). The extraordinary thermostability of CtCel7 gives this enzyme a top rank among previously reported bifunctional GHs, such as HcCel5 and XynA (Ghatge et al., 2014; Wang et al., 2019), which is likely associated with its relatively high number of disulfide bonds (Figure 1B) and glycosylation (Figures 1B, 3). Normally, thermostable enzymes tend to contain additional disulfide bonds to limit the motion of the random coil and reduce the conformational entropy of the unfolded polypeptide, especially when these S-S bridges are located at the enzyme’s surface and far from the catalytic center (Liu et al., 2016; Li G. et al., 2018). Indeed, 10 disulfide bonds are present in CtCel7, and this number is higher than the number of disulfide bonds found in other GH7 thermostable cellobiohydrolases, including HgtCel7A (nine disulfide bonds) (Momeni et al., 2013) and GcaCel7A (eight disulfide bonds) (Borisova et al., 2015). Additionally, previous studies have demonstrated that glycosylation is conducive to preserving protein stability as a result of more extended conformations which provide higher free energy of the unfolded polypeptide (Helenius and Aebi, 2004; Shental-Bechor and Levy, 2008). However, the origin of the extraordinary thermostability of CtCel7 is complex, and additional in-depth studies focusing on the biological rationale of thermostable enzymes should be performed.

### Effects of Metal Ions and Reagents on Enzymatic Activity

In the industrial processing of straw, heavy metal ions are inevitably present in productive processes because straw is a natural biosorbent with a quite complex adsorption mechanism through surface precipitation, ion-exchange and complexation (Farooq et al., 2010). Besides, in papermaking, a large amount of calcium ions and magnesium ions are employed to enhance opacity in filling and coating operations (Boyce and Walsh, 2015). Thus, a considerable metal insensitivity to metal ions is an advantageous characteristic for enzymatic biodegradation of cellulosic materials in various practices, including bioethanol preparation, waste biodegradation, feed additive production and textile industry. CtCel7 displayed substantial abilities to withstand most tested metal ions, with the exception of Cu^2+^ and Fe^3+^, which completely suppressed enzymatic hydrolysis at a concentration of 5 mM. Ni^2+^ and Cr^3+^ were found to have beneficial effects on cellulase activity. Conversely, the xylanase activity was decreased in the present of Ni^2+^ and Cr^3+^. In addition, xylanase activity was reduced by nearly half in the presence of Zn^2+^ ions at a low concentration (1 mM) and completely inactivated when the concentration was increased to 5 Mm. However, Zn^2+^ rarely impacted the hydrolysis of glucan (Table 2). The discrepant influence of one metal ion on enzyme activities is probably attributed to the different catalytic mechanisms for hydrolyzing glucan and xylan. Actually, there is not a fairly uniform pattern for the effect of metal ions on cellulase and xylanase activities (Chang et al., 2011; Ko et al., 2011; Peng et al., 2016; Li W. et al., 2018). More details regarding the exact mechanism involved in enhanced properties with metal ions should be elucidated by subsequent experiments, including resolving the 3D structure and investigating additional rational protein designs.

**TABLE 2:** Effect of metal ions on bifunctional CtCel7 activities.

	Relative activity (%)			
	Glucan		Xylan	
Metal ions	1 mM	5 mM	1 mM	5 mM
Control	100	100	100	100
Mn^2+^	82.1 ± 2.7	109.8 ± 3.6	59.3 ± 4.0	73.0 ± 3.9
Ca^2+^	82.6 ± 2.8	93.9 ± 6.7	88.5 ± 5.9	94.8 ± 7.4
Mg^2+^	82.9 ± 8.0	68.3 ± 1.3	101.0 ± 1.0	84.5 ± 9.1
Co^2+^	95.8 ± 4.7	68.8 ± 8.1	78.2 ± 7.5	57.4 ± 7.2
Sr^2+^	88.1 ± 6.6	87.4 ± 2.1	93.0 ± 5.8	97.0 ± 4.0
Ni^2+^	116.7 ± 7.2	125.6 ± 5.2	87.7 ± 6.0	69.1 ± 2.2
Zn^2+^	94.1 ± 4.9	104.2 ± 2.7	47.3 ± 4.8	*ND*
Cr^3+^	134.3 ± 5.6	107.1 ± 3.7	71.1 ± 2.9	55.1 ± 6.3
Al^3+^	92.5 ± 5.0	94.0 ± 4.4	95.3 ± 2.1	77.2 ± 6.8
Cu^2+^	31.9 ± 5.7	ND	ND	ND
Fe^3+^	21.7 ± 2.8	ND	28.7 ± 7.0	ND

*The activities without supplementation of metal ions (control) were defined as 100%. ND represents that no activity was detected. Values are the means ± SD of three replicates.*

One of the important strategies for modulating enzyme activity is the use of additives to affect their microenvironment in the biochemical industry (Kirk et al., 2002). As shown in Table 3, the two inherent hydrolytic activities of CtCel7 exerted similar tendencies in the presence of chemical reagents. Notably, EDTA and SDS severely compromised the efficiency of the bifunctional activities of CtCel7. Similar results were detected for a GH7 endo-β-1,4-glucanase (Cel7A) (Liu et al., 2015), a GH10 xylanase (Xyln-SH1) (Cheng et al., 2012) and a GH5 bifunctional cellulase-xylanase (CbGH5) (Tan et al., 2018). The beneficial effect of Tween-20, Triton X-100 and PEG-600 on enzyme activity may be attributed to the effect of surfactant binding on enzyme secondary and tertiary structure that results in a more flexible conformation (Delorme et al., 2011; Holmberg, 2018). Alternatively, in the case of appropriate surfactants, the reaction environment will be optimized and subsequently make the enzyme suitable for use in industrial processes (Goldfeder and Fishman, 2014). The insensitivity of CtCel7 to most of the tested metal ions (Table 2) and chemical reagents (Table 3) is a notable industrial advantage (Yang et al., 2017).

**TABLE 3:** Effects of chemical reagents on bifunctional CtCel7 activities.

	Relative activity (%)	
Chemicals	Glucan	Xylan
Control	100	100
0.1% Tween-20	107.4 ± 3.9	110.3 ± 5.2
1% Triton X-100	112.6 ± 3.6	106.8 ± 3.8
1% PEG-600	115.8 ± 2.8	94.1 ± 3.0
1% methanol	83.6 ± 2.4	86.0 ± 3.9
1% ethanol	87.4 ± 2.5	87.5 ± 4.6
1% isopropanol	80.6 ± 1.7	86.8 ± 2.7
1 mM EDTA	70.7 ± 6.2	62.9 ± 2.5
5 mM EDTA	54.1 ± 3.4	49.8 ± 3.3
1% SDS	ND	ND

*The activities without supplementation of reagents (control) were defined as 100%. ND represents that no activity was detected. Values are the means ± SD of three replicates.*

### Substrate Specificity of CtCel7

The quantification of the substrate preference of the enzyme based on the presence of different glycosidic bonds demonstrated that the bifunctional CtCel7 was most adept in the microcrystalline cellulose depolymerization of PASC and filter paper with values of 2.54 and 2.16 IU/mg, respectively, which were relatively higher than those obtained with the commercial cellobiohydrolase TlCBHI and the commercial xylanase TlXyn. The hydrolysis of crystalline cellulose is the fundamental substrate preference of CBHs and is a central step in cellulose material degradation in natural and industrial settings (Taylor et al., 2018). In addition, CtCel7 exhibited efficient hydrolysis of soluble cellulosic substrates (glucan and CMC-Na), xylan and lichenin. No activity was observed with other polysaccharide substrates (Table 4). These results revealed that CtCel7 has a remarkable capability to specifically degrade a variety of polysaccharide substrates with the typical β-1,4-linkage.

**TABLE 4:** Substrate specificity of bifunctional CtCel7.

		Specific Activity (IU/mg)		
Substrates	Main linkage types	CtCel7	TlCBHI	TlXyn
PASC	β-1,4-glucan	2.54 ± 0.15	1.23 ± 0.16	1.32 ± 0.17
filter paper	β-1,4-glucan	2.16 ± 0.39	1.11 ± 0.24	1.74 ± 0.31
glucan	β-1,4-glucan	1.94 ± 0.16	0.95 ± 0.08	1.58 ± 0.05
CMC-Na	β-1,4-glucan	0.48 ± 0.07	0.62 ± 0.13	0.44 ± 0.03
xylan	β-1,4-xylan	1.49 ± 0.11	0.21 ± 0.04	2.45 ± 0.24
lichenin	β-1,3/1,4-glucan	1.30 ± 0.06	0.79 ± 0.02	1.87 ± 0.02
mannan	β-1,2/1,3-glucosamine	ND	ND	ND
chitin	β-1,4-glucopyranose	ND	ND	ND
arabinogalactan	β-1,3/6-galactose	ND	ND	ND
sucrose	α-1,2-glucan	ND	ND	ND
amylose	α-1,4-glucan	ND	ND	ND
amylopectin	α-1,6-glucan	ND	ND	ND

*ND indicates that no activity was detected. Values are the means ± SD of three replicates.*

### Kinetic Parameters of CtCel7

The Michaelis-Menten kinetic constants of CtCel7 were evaluated using glucan and xylan as the substrates. As illustrated in Table 5, the Km values for glucan and xylan hydrolysis were 4.06 and 4.91 mg/ml, respectively, which suggest that CtCel7 exhibits higher affinity for glucan than for xylan. Moreover, the preferable reaction velocity (Vmax), turnover rate (kcat) and catalytic efficiency (kcat/Km) against glucan compared with those against xylan indicated that the enzyme induced superior catalytic hydrolysis of glucan, which was in accordance with the specific activities of bifunctional CtCel7 on lignocellulosic materials.

**TABLE 5:** Kinetic parameters of bifunctional CtCel7.

	Km	Vmax	kcat	kcat/Km
Substrates	(mg/ml)	(μg/min/ml)	(s^–1^)	(μl/s/mg)
Glucan	4.06 ± 1.40	18.45 ± 3.52	1.54 ± 0.29	391.34 ± 52.74
Xylan	4.91 ± 0.81	10.78 ± 0.63	0.90 ± 0.05	184.94 ± 18.35

*Values are the means ± SD of three replicates.*

### Hydrolytic Product Analysis of CtCel7

**FIGURE 5:**
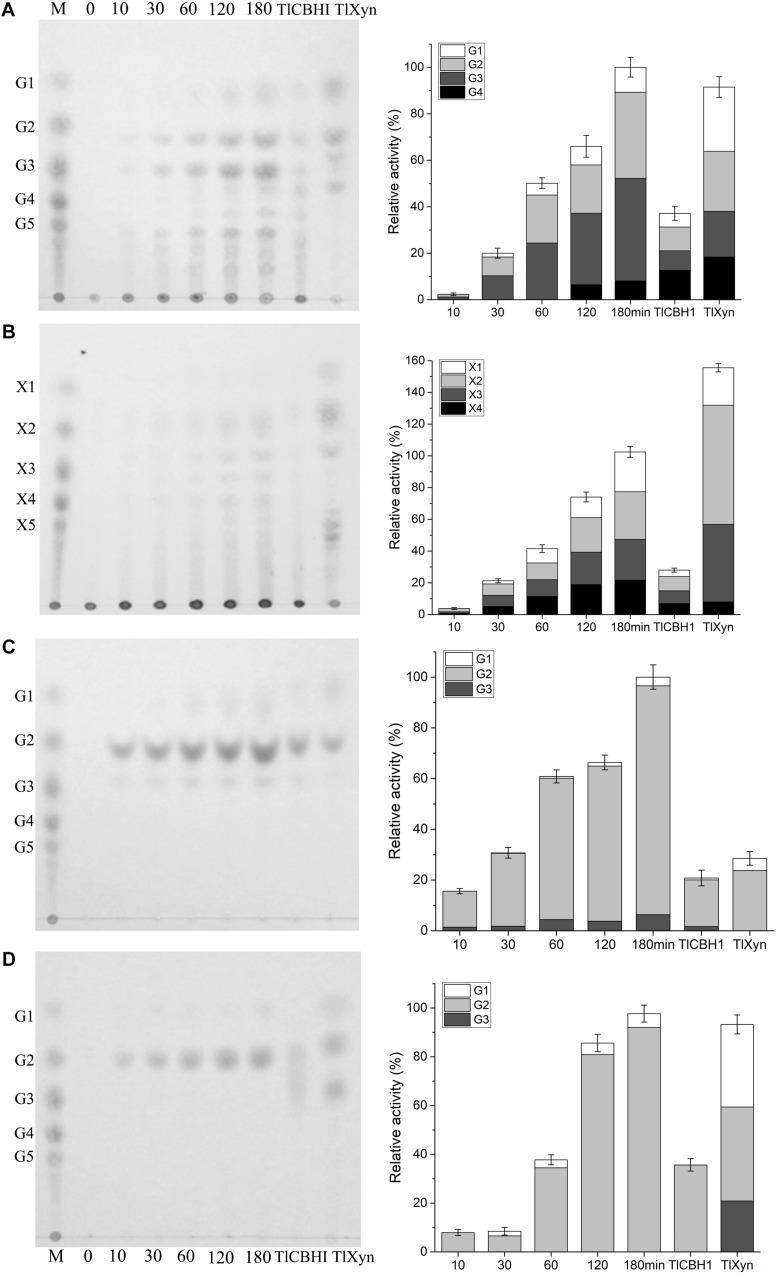
Thin-layer chromatography of the hydrolysis products of CtCel7 against glucan **(A)**, xylan **(B)**, PASC **(C)** and filter paper **(D)** compared with those obtained with the commercial cellobiohydrolase TlCBH1 and the commercial xylanase TlXyn. The hydrolysis profiles of TlCBH1 and TlXyn were detected after 180 min of incubation at their respective optimal reaction conditions. Lane M, the oligosaccharide standard. G1-G6 and X1-X6 represent the degree of polymerization of cellulose and xylan, respectively. The relative proportions of the products were calculated through a gray-value analysis of the TLC results using ImageJ software. The values are the means ± SDs from three replicates.

To determine whether CtCel7 has an endo- or exocatalytic action, the enzymolysis products of glucan, xylan, PASC, and filter paper were determined in a time-dependent manner via TLC and compared with those obtained with commercial TlCBHI and TlXyn (Figure 5). During the sequential degradation process, the amount of oligosaccharide products released from glucan and xylan gradually became increasingly distinct, and the final products were mainly monosaccharides, disaccharides and trisaccharides, which aligned with the hydrolytic profile of TlXyn. Nevertheless, the major hydrolysis product of insoluble cellulosic PASC and filter paper was cellobiose, which was similar to the hydrolytic profile of TlCBHI. These results further confirmed that bifunctional CtCel7 possesses both cellobiohydrolase and xylanase activities.

The generation of soluble oligosaccharides from lignocellulose is an essential premise in practical production. Notably, CtCel7 was able to continuously saccharify cellulosic substrates and xylan to produce high percentages of cello-oligosaccharides and xylo-oligosaccharides (Figure 5), respectively, which are valuable feedstocks for various biological and biochemical applications (Han X. et al., 2018).

### Synergistic Effect of CtCel7 and Commercial Cellulase on Pretreated Wheat Straw Hydrolysis

**FIGURE 6:**
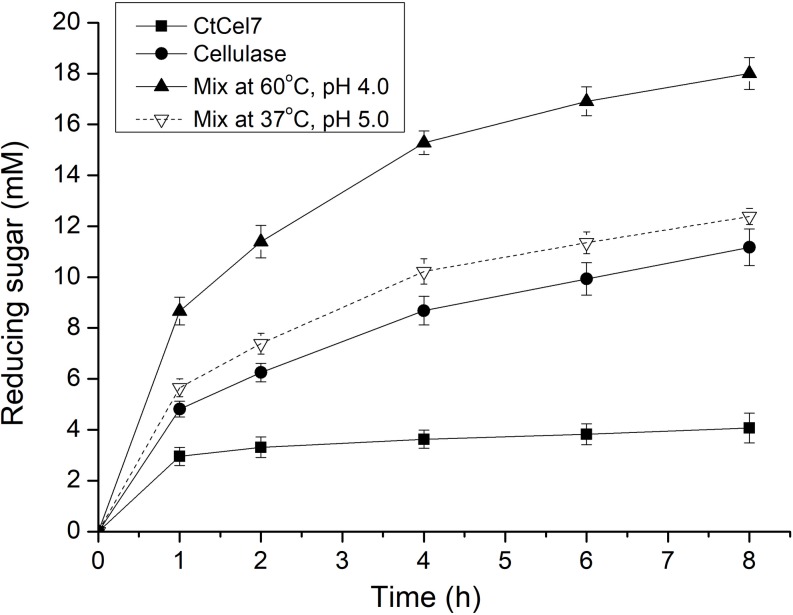
Synergistic hydrolysis of pretreated wheat straw by CtCel7 and a commercial *T. reesei* cellulase cocktail. The synergistic hydrolysis induced by CtCel7 and the cellulase cocktail was performed under the optimal reaction conditions of both enzymes. Each reaction initially contained 12 mg of biomass and 80 μg of CtCel7 and/or the Cellulase cocktail. The values are the means ± SDs from three replicates.

Wheat straw is an abundant agricultural residue and is applied as a feedstock for the production of fodder, biofuels and other bioproducts. To further investigate the relevance of the enzyme in the industrial context, the cooperative action of CtCel7 with commercial cellulase on the degradation of pretreated wheat straw was investigated (Figure 6), and the results showed that CtCel7 hydrolysis was marginally increased throughout the process. In contrast, the reducing sugar concentrations were increased by cellulase and the synergistic combination. After an 8-hour reaction period, the concentration of released reducing sugars produced by CtCel7 alone was low (3.8 mM), but the mixture of CtCel7 and cellulase (reaction at 60°C and pH 4.0) produced a significantly higher amount of reducing sugars (18.3 mM) than those produced by cellulase alone (11.2 mM) and by the mixture that was preincubated at 37°C and pH 5.0 (12.4 mM). These data indicated that CtCel7 exerts a substantial synergistic effect with cellulase on PWS hydrolysis at high temperatures.

Xylan, the primary constituent of hemicellulose, acts as the articulated skeleton and alternates with cellulose fibrils to restrict enzymatic degradation of its structural polysaccharides (Grantham et al., 2017). CtCel7 might efficiently attack xylan to substantially facilitate access to cellulase and the deconstruction of lignocellulose (Long et al., 2018). A similar result was observed for a synergistic effect of cellulase-xylanase that was more effective than either of the single enzyme treatment on natural lignocellulosic substrates, such as corncob, corn stover, and rice straw (Song et al., 2016). Alternatively, CtCel7 may serve as an efficient cellobiohydrolase by acting in combination with the cellulase cocktail to further enhance the hydrolysis effect on the abundant crystalline cellulose in straw and thus accelerate the degradation process compared with that achieved with cellulase alone.

## Conclusion

A novel GH7 family glycoside hydrolase (CtCel7) from thermophilic *C. thermophilum*, which exhibits prominent bifunctional cellobiohydrolase-xylanase activity and considerable thermostability, was identified and characterized. CtCel7 is able to effectively hydrolyze cellulosic substrates into fermentable sugars, which are important feedstocks in food, biofuel and biochemical production. In addition, the hydrolysis effect of the commercial cellulase cocktail on pretreated wheat straw was greatly improved by its combination with CtCel7. The excellent properties of CtCel7 make it a potential biocatalyst for the conversion of biomass in practical biotechnological applications.

## Data Availability Statement

The datasets analyzed in this article are not publicly available. Requests to access the datasets should be directed to CH, hanch87@163.com.

## Author Contributions

CH, RY, YS, ML, and LZ performed the major experiments, analyzed the data, and contributed to the manuscript. CH supervised the project and the writing of the manuscript which was revised by DL. CH and DL coordinated the final version of the manuscript. All authors have read and approved the final manuscript.

## Conflict of Interest

The authors declare that the research was conducted in the absence of any commercial or financial relationships that could be construed as a potential conflict of interest.

## Footnotes

1 http://ct.bork.embl.de/

2 http://web.expasy.org/protparam/

3 http://www.cbs.dtu.dk/services/SignalP/

4 http://www.cbs.dtu.dk/services/NetNGlyc/

5 http://www.cbs.dtu.dk/services/NetOGlyc/

6 https://blast.ncbi.nlm.nih.gov/Blast.cgi
