# Supplementary material for: Identification and Characterization of a Novel Hyperthermostable Bifunctional Cellobiohydrolase- Xylanase Enzyme for Synergistic Effect With Commercial Cellulase on Pretreated Wheat Straw Degradation
Source: Front Bioeng Biotechnol. 2020 Apr 9;8:296. doi: 10.3389/fbioe.2020.00296 (PMC7160368; doi:10.3389/fbioe.2020.00296)

**Supplementary data**

**Figure Legend**

**FIGURE S1** MALDI-TOF/TOF analysis of purified protein CtCel7. For mass spectrum analyze, the purified protein was excised from SDS-PAGE gel and subjected to trypsin digestion. Then the resultant peptides were analyzed by MALDI TOF/TOF MS using 5800 MALDI TOF/TOFTM Analyzer (AB Sciex, Massachusetts, USA). The acquired mass spectra of peptides were search for similarities using Mascot software (Matrix Science, Boston, MA, USA).


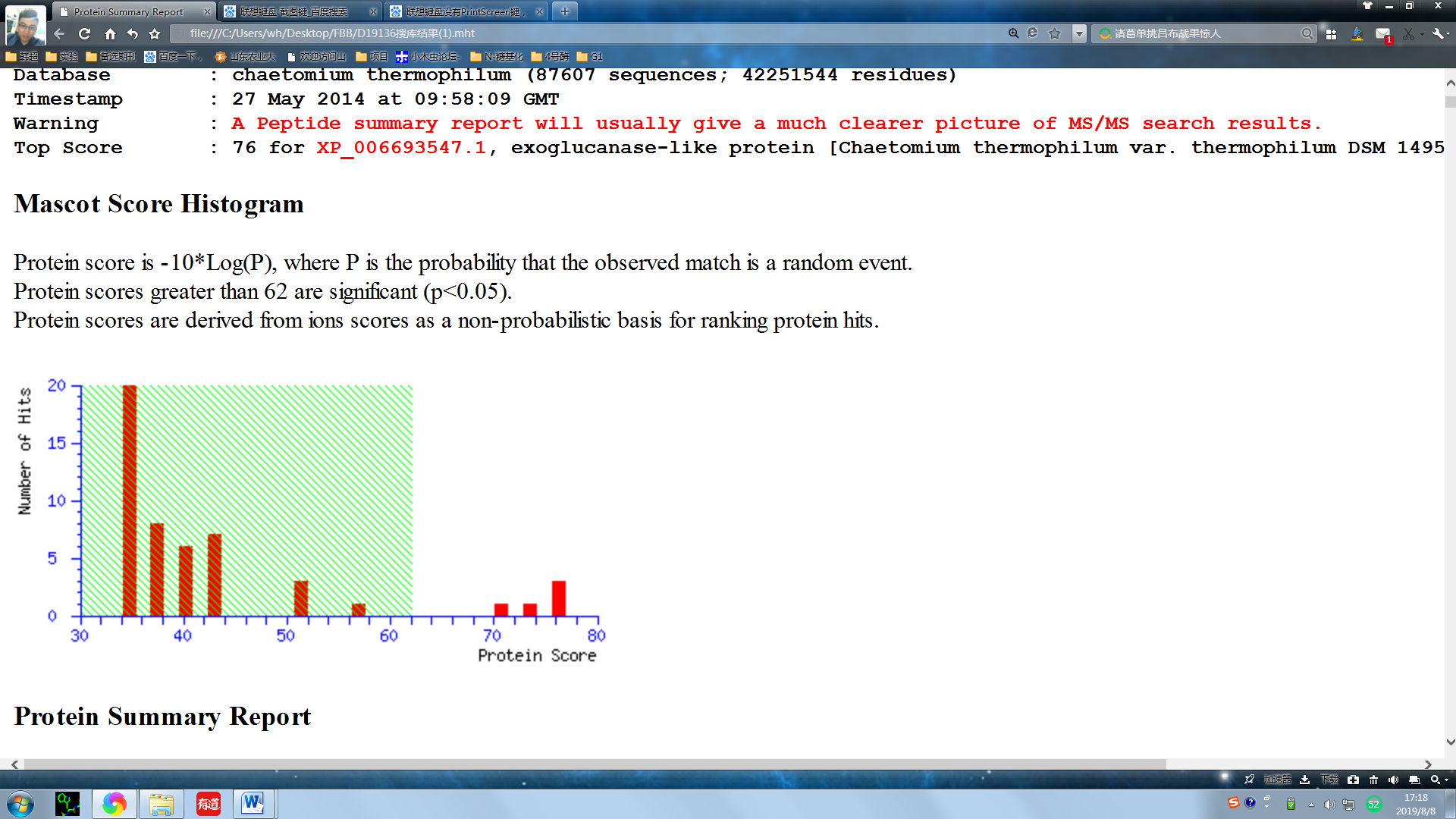


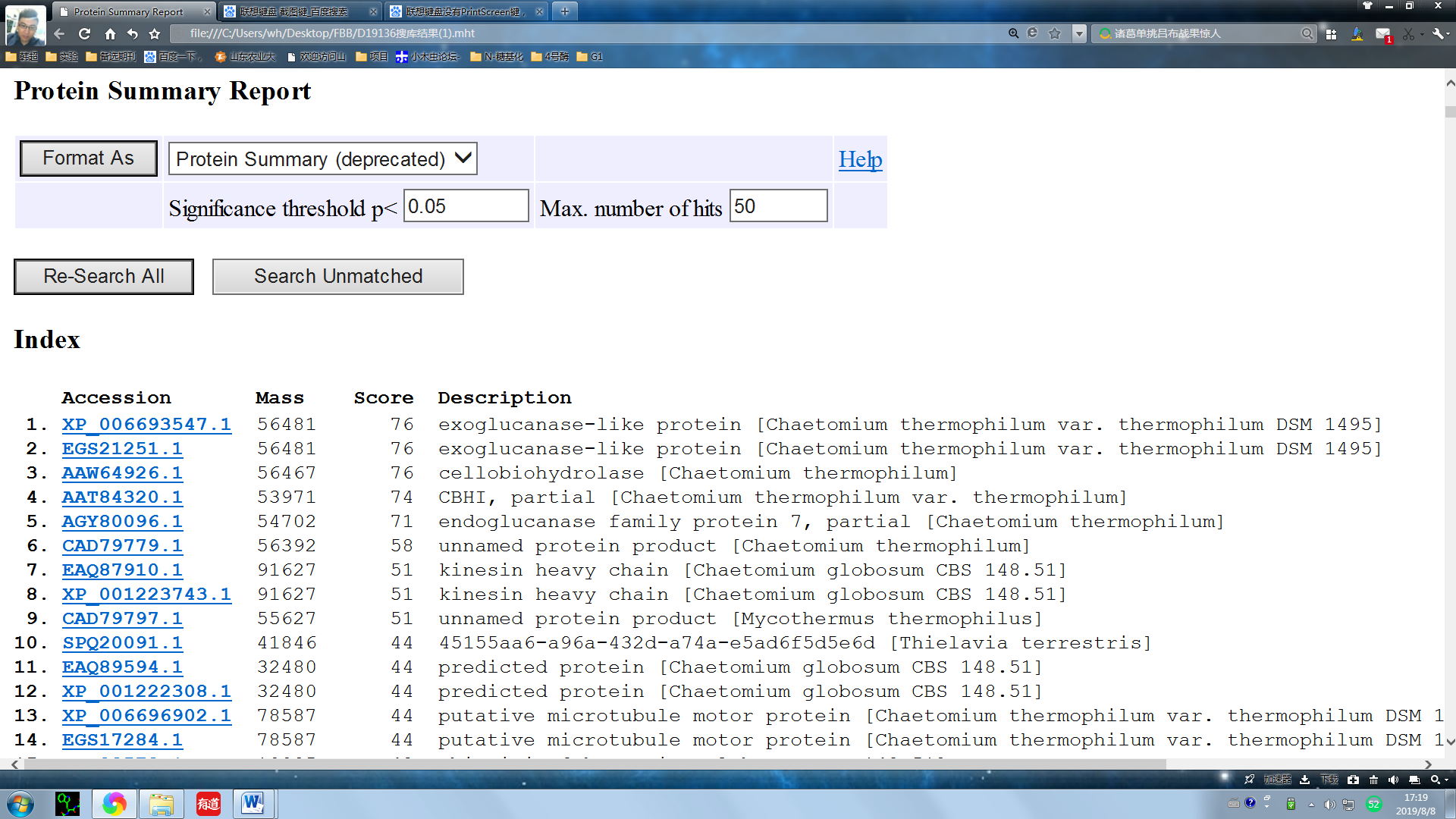


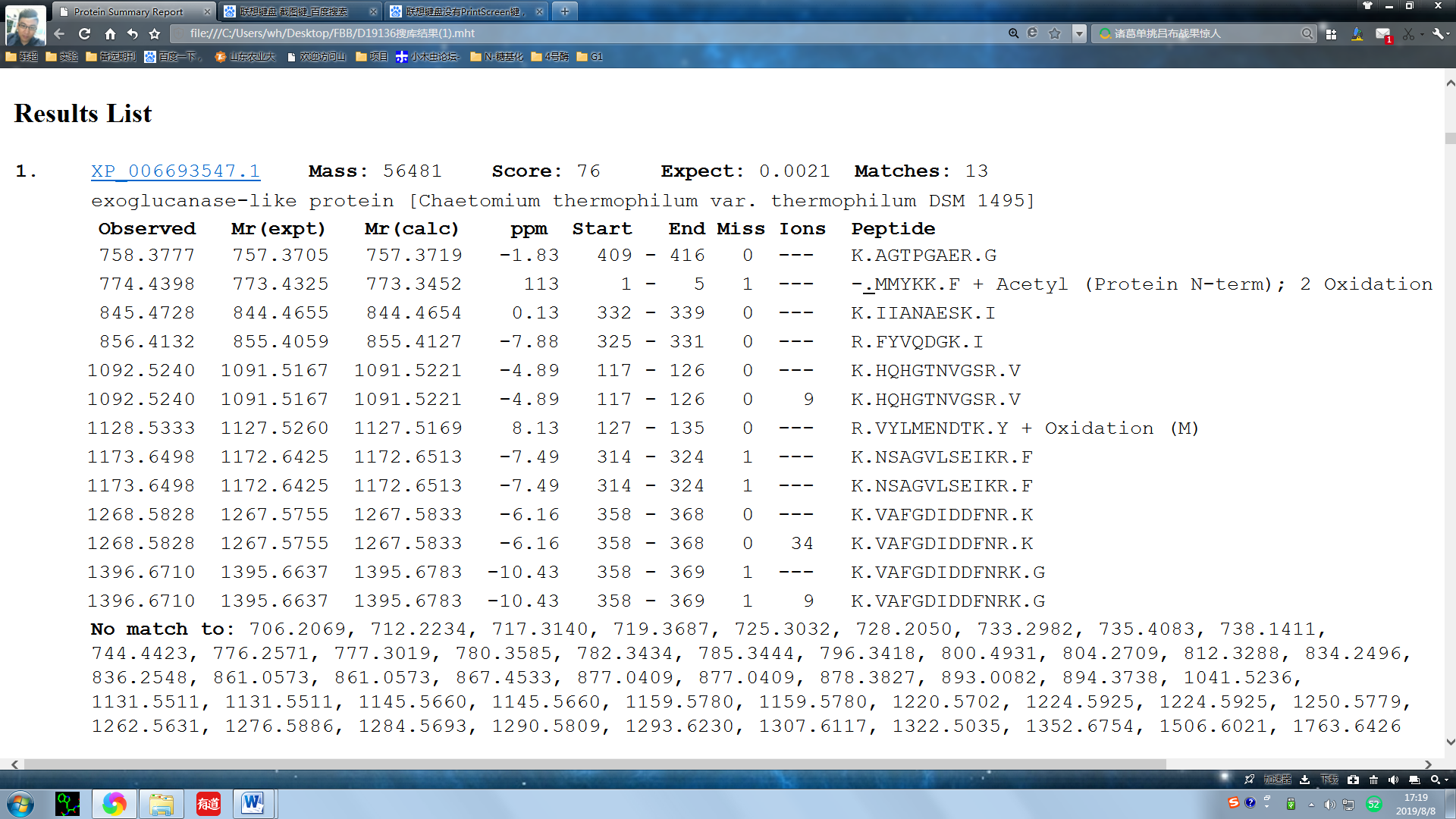

Supplement: Supplementary file 1 [file Table_1.DOCX]
